# Mature cystic teratoma with co-existent mucinous cystadenocarcinoma: describing a diagnostic challenge—a case report

**DOI:** 10.1186/s13256-024-04544-w

**Published:** 2024-05-05

**Authors:** Mahboobeh Chahkandi, Farnaz Mozayani, Ali Fanoodi, Amir Reza Bina, Amir Reza Ebrahimian

**Affiliations:** 1 Department of Pathology, School of Medicine, Birjand University of Medical Sciences, Birjand, Iran; 2 Student Research Committee, Birjand University of Medical Sciences, Birjand, Iran; 3 Cellular and Molecular Research Center, Birjand University of Medical Sciences, Birjand, Iran

**Keywords:** Dermoid cyst, Cystadenocarcinoma, Mucinous, Collision tumor, Combined tumor, Case report

## Abstract

**Background:**

Mature cystic teratoma co-existing with a mucinous cystadenocarcinoma is a rare tumor that few cases have been reported until now. In these cases, either a benign teratoma is malignantly transformed into adenocarcinoma or a collision tumor is formed between a mature cystic teratoma and a mucinous tumor, which is either primarily originated from epithelial-stromal surface of the ovary, or secondary to a primary gastrointestinal tract tumor. The significance of individualizing the two tumors has a remarkable effect on further therapeutic management.

**Case presentation:**

In this case, a mature cystic teratoma is co-existed with a mucinous cystadenocarcinoma in the same ovary in a 33-year-old Iranian female. Computed Tomography (CT) Scan with additional contrast of the left ovarian mass suggested a teratoma, whereas examination of resected ovarian mass reported an adenocarcinoma with a cystic teratoma. A dermoid cyst with another multi-septate cystic lesion including mucoid material was revealed in the gross examination of the surgical specimen. Histopathological examination revealed a mature cystic teratoma in association with a well-differentiated mucinous cystadenocarcinoma. The latter showed a CK7−/CK20 + immune profile. Due to the lack of clinical, radiological, and biochemical discoveries attributed to a primary lower gastrointestinal tract tumor, the immune profile proposed the chance of adenocarcinomatous transformation of a benign teratoma.

**Conclusions:**

This case shows the significance of large sampling, precise recording of the gross aspects, histopathological examination, immunohistochemical analysis, and the help of radiological and clinical results to correctly diagnose uncommon tumors.

## Introduction

Mature cystic teratoma co-existing with a mucinous cystadenocarcinoma is a rare tumor that few cases have been reported till now [1]. In these cases, either a benign teratoma is malignantly transformed into adenocarcinoma or a collision tumor formation between a mature cystic teratoma and a mucinous tumor, either primarily originated from epithelial-stromal surface of the ovary, or secondary to a primary gastrointestinal tract tumor [1]. The significance of individualizing the two entities has remarkable effects on further therapeutic management [1].

Mature cystic teratomas, which can occur at all ages, are often found in young adults and children. Benign cases of teratomas are less likely (1–2%) to become malignant [2, 3]. Also, they are most likely to develop to squamous cell carcinomas (SCC) [3, 4]. However, 7% of reported malignant cases are adenocarcinomas [5]. Mucinous tumors are correlated with 2–11% of cases with mature cystic teratoma [6], which might become cystadenoma, borderline, or even malignant; the last composition is clearly infrequent.

It should be noted that this case report has been written using the CARE reporting guidelines [7], and the informed consent was obtained from the patient.

## Case presentation

A 33-year-old Iranian woman with a history of four Natural Vaginal Deliveries (NVDs) was admitted for evaluation of a 2-month delay in her menstrual cycle in 10th, June 2020. The patient had no special past medical history (including any malignancies in the breast, gastrointestinal tract, or other organs). During the admission, the patient’s vital signs were stable; however, in physical examination, one hard mass in right hypogastric region was found.

In the same day, lab tests showed mild normocytic normochromic anemia, while liver and kidney function tests, along with the urine culture test were normal. Tumor markers were checked for the patient; CA19-9 was 104.9 U/L (reference range: 0–33 U/L), and CA125 was 21.3 U/L (reference range: < 35 U/L).

**Fig. 1:**
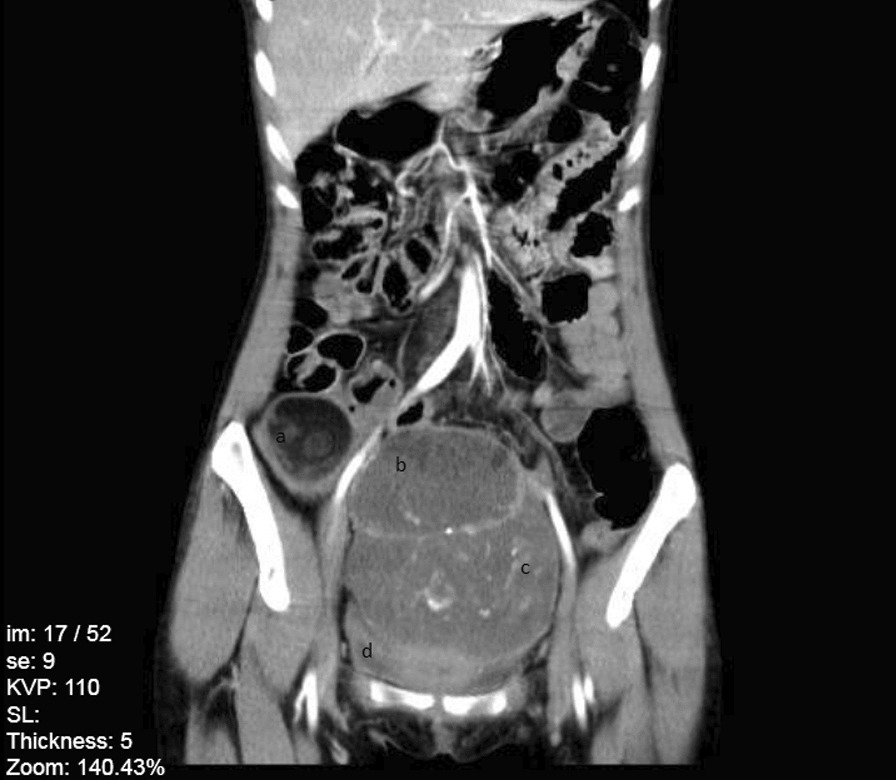
Abdominal CT-Scan with intravenous (IV) contrast, coronal view. **a** Right ovarian mass, **b** Uterine, **c** Left Ovarian mass, **d** Bladder (empty)

**Fig. 2:**
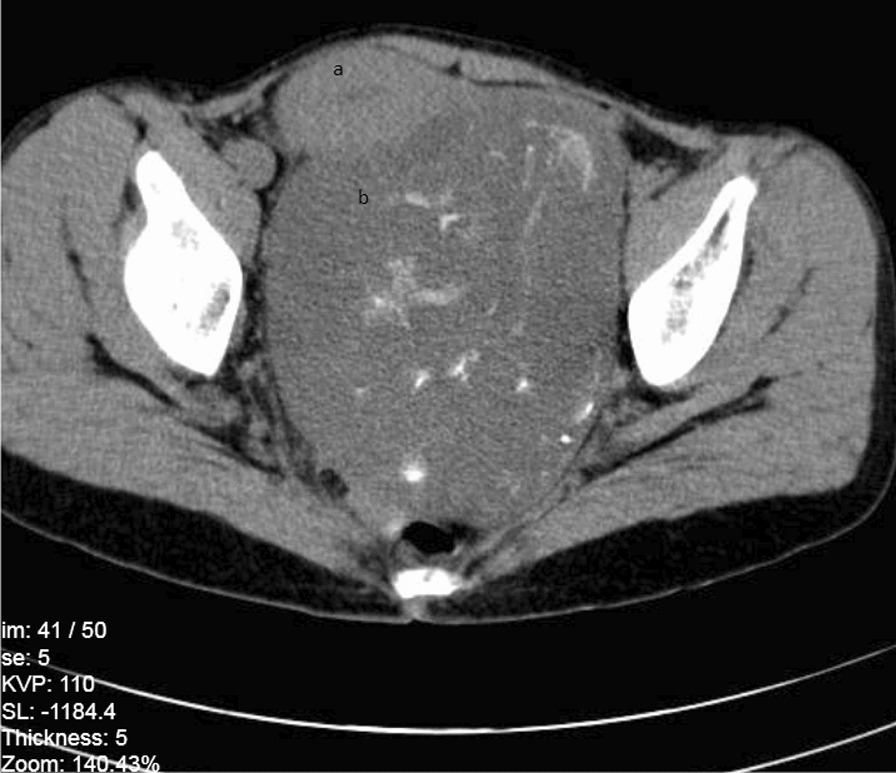
Abdominal CT-Scan without IV contrast, axial view. **a** Uterine, **b** Left ovarian mass

**Fig. 3:**
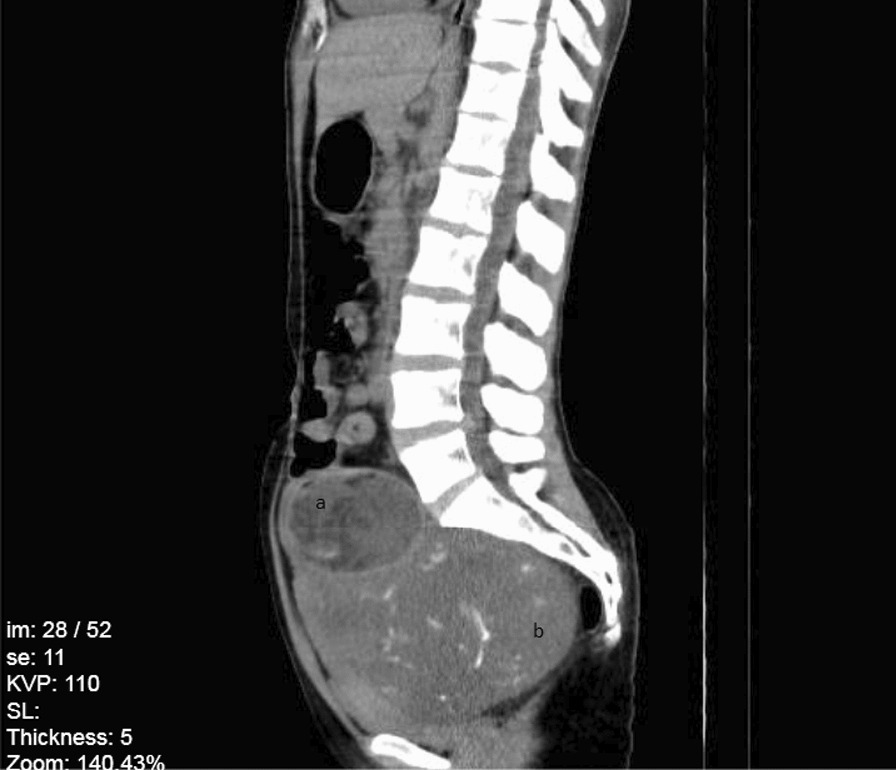
Abdominal CT-Scan without IV contrast, sagittal view. **a** Uterine, **b** Left Ovarian mass

In the next day, transvaginal ultrasound revealed a heterogenous mass in posterior cul-de-sac and retrovesical area, which led to the displacement of the urinary bladder and uterus. A mass lesion with dimensions of 154*137*111 mm with mixed density containing cystic, solid, and fat components along with the multiple foci of calcifications were found in right paracolic gutter in Computed Tomography Scan (CT-Scan) in 13th, June 2020. Due to the presentation of a mixed density element on one side and a heterogenous solid element on the other side of the lesion, probability of collision tumor and immature teratoma was proposed. CT-Scan stereotypes showed a right ovarian mass as well as a left ovarian mass which caused a mass effect on uterine (Figs. 1, 2, 3). Since the laboratory data suggested a mucinous cyst, an appendectomy was also suggested. On 14th, June 2020, the patient underwent left salpingo-oophorectomy, right ovarian cystectomy, omentectomy, and appendectomy; however, uterus and right ovary were preserved. The histological evaluation of appendix was reported normal. A colonoscopy was performed in to rule out gastrointestinal tract metastasis, which later appeared to be normal.

Gross examination of the previously-cut specimen, labeled as left ovarian cyst, on 14th, June 2020 revealed round gray mass with smooth external surface measuring 130*120*120 mm with two lesions with clear margins involving the left ovary. The larger one measuring 120*100*45 mm with solid and cystic cut surface contained thick mucoid material and necrotic tissue. Adjacent cystic space contained sticky yellow material and tufts of hair measuring 70*60*55 mm and showed one elastic projection measuring 30*15*10 mm. The right ovarian cyst was composed of previously-opened multilocular cyst measuring 65*35*35 mm, containing tufts of hair and yellow material without solid component. Focal elastic projection measuring 15*10*5 mm was also observed. The omentum, left fallopian tube, and appendix seemed to be unremarkable.

**Fig. 4:**
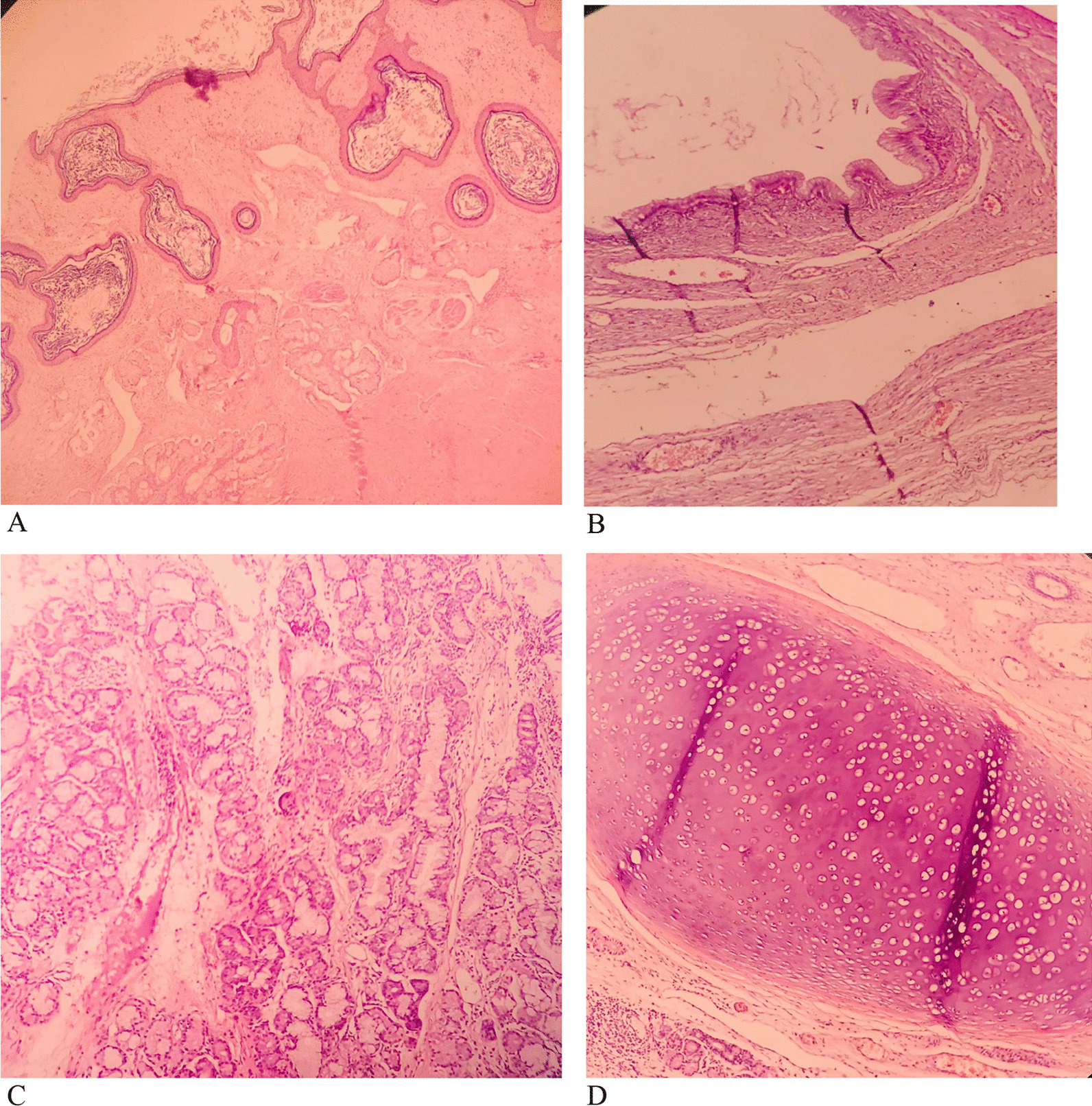
Mature teratoma component of tumor includes skin (**A**), gastrointestinal mucosa (**B**), salivary glands (**C**), and cartilage (**D**) (H&E, 100×)

**Fig. 5:**
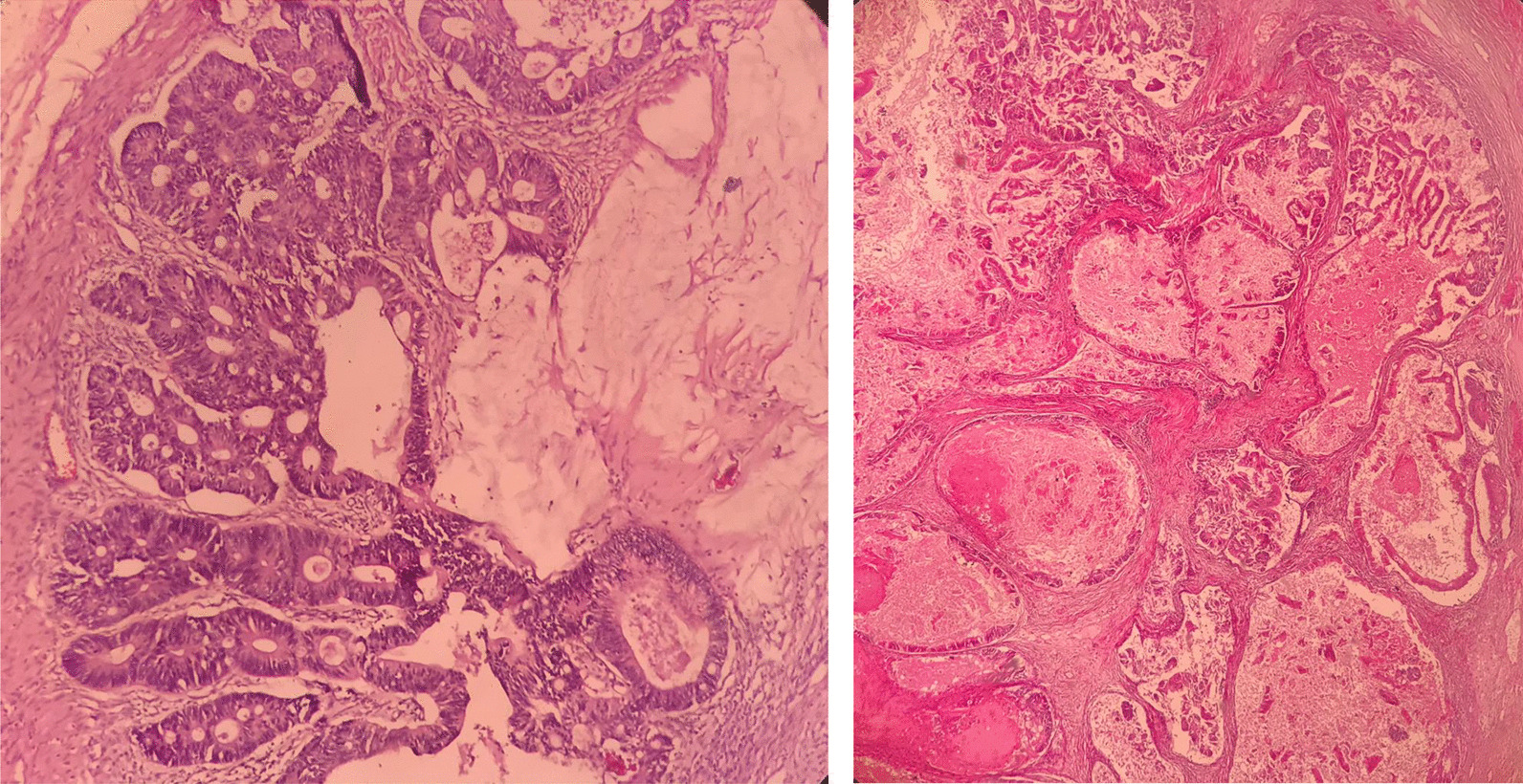
Mucinous adenocarcinoma component (H&E, 100×)

Microscopic slides of smaller cystic lesion showed mature teratoma composed of normal skin tissue with its appendages like hair follicles, sebaceous glands, and subcutaneous adipose tissue. Respiratory mucosa, gastrointestinal mucosa (mostly of colon type), salivary glands, and mature cartilage tissue were also observed (Fig. 4). The slides of larger solid cystic mass showed extensive necrosis, back-to-back variable-sized glandular structures, cribriform, and fused glands lined with stratified mucinous columnar epithelial cells, which included pencil-like atypical nuclei, scattered nucleoli, and exhibited prominent mitosis (Fig. 5).

A 6-month follow-up of the patient by checking tumor markers appeared to be normal, and there were no signs of tumor markers rising.

## Discussion

**Fig. 6:**
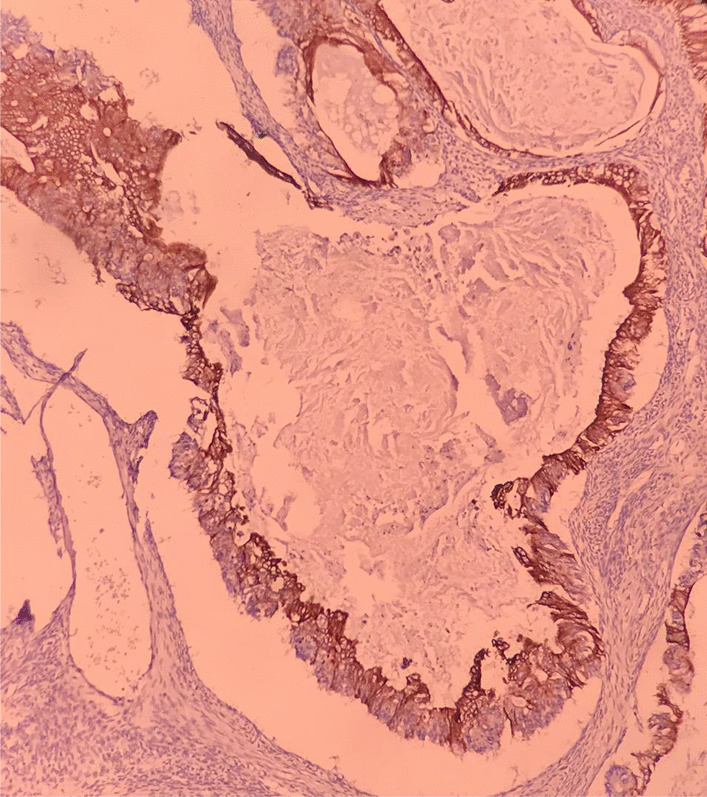
CK20 positivity in adenocarcinoma component (Immunohistochemistry, 100×)

**Fig. 7:**
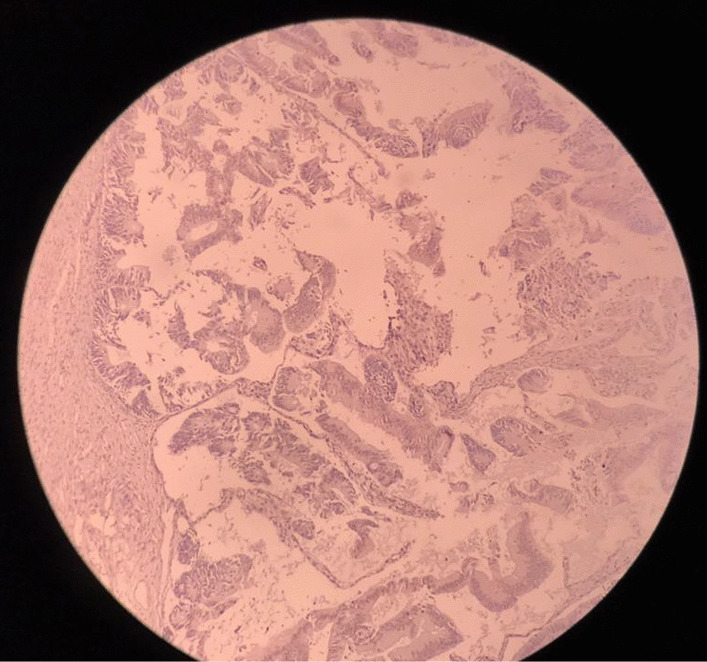
CK7 negativity in adenocarcinoma component (Immunohistochemistry, 100×)

It has been formerly reported that many teratomas create collision tumors in the ovary in combination with serous adenocarcinoma, steroid cell tumors, and granulosa cell tumor [8]. The most important criterion to distinguish collision tumor is the lack of histological mixture at the junction between the two tumor structures [9]. Although, based on imaging examination, a collision tumor was suggested in this case, histological examination did not show normal ovarian parenchyma between the two tumor lesions to be diagnosed as a collision tumor. Variable expression of CK20 with diffuse CK7 positivity are shown in the majority of primary ovarian mucinous tumors of surface epithelial-stromal origin [10]. In this case, a CK20 + /CK7 − immune profile was shown in the immunohistochemical analysis in the mucinous cystadenocarcinoma component (Figs. 6 and 7). Primary metastases from lower gastrointestinal tract such as colorectum or the appendix, along with the mucinous neoplasm originated from the tissue type of lower gastrointestinal tract existing in the ovarian teratoma, can describe such immune profile.

A second malignant neoplasm was not manifested by radiological and pre-operative clinical evaluation in the upper or lower gastrointestinal tract or any other organs. Serological evaluations revealed high levels of CA19-9; however, CA-125 was reported within the normal range. No evidence of tumor was seen in the surgical specimen of the appendix. Hence, a mucinous cystadenocarcinoma of the intestinal-type deriving from a mature cystic teratoma was likely to be the diagnosis. No recurrence or secondary neoplasm was revealed in a 22-months post-operative follow-up period in the patient, which confirms our diagnosis. There was no certain proof of a primary cause more than a metastasis in previous studies in similar patients [11–14]. In a recent case of mucinous cystadenocarcinoma deriving from a mature cystic teratoma, due to the lack of recurrence for a 6-month follow-up period and CK7 positivity, the adenocarcinoma was considered as a primary etiology. However, the follow-up period was 22 months in the present case report.

Evaluation of the resected surgical specimen of the appendix ruled out the primary mucinous neoplasm. No radiological or serological proof of a secondary tumor was observed. CK20 + /CK7 − profile in immunohistochemical analysis and an intermediate transitional zone between the benign and malignant components described by the histopathological evaluation of the ovarian mass were strong proofs to confirm our diagnosis. It is possible that an additional neoplasm occurs in the same ovary, given the large variety of endodermal, ectodermal, and mesodermal tissues that a teratoma may be correlated with. In a study on 44 mucinous ovarian tumors correlated with mature cystic teratoma by Vang *et al*., only 6 cases had carcinomatous transformation, which all of them had pseudomyxoma ovarii [10]. In this study, the attendance of the pseudomyxoma ovarii was remarkably correlated with a CK20 + /CK7 − immune profile. A secondary ovarian tumor arising from a primary malignancy of the lower gastrointestinal tract can also be proposed by a CK20 + /CK7 − immune profile. Nonetheless, additional aspects of metastasis such as extra-pelvic spread, multi-nodularity, bilateral involvement, and hilus involvement of the tumor are important clues as complete clinical evaluation and follow-up failing to find a non-ovarian origin of mucinous neoplasm in another place [10].

## Conclusions

This case shows the significance of large sampling, precise recording of the gross aspects, histopathological examination, immunohistochemical analysis, and the help of radiological and clinical results to correctly diagnose uncommon tumors.

## Data Availability

The datasets used during the current study are available from the corresponding author on reasonable request.
